# Lipidomic and Transcriptomic Basis of Lysosomal Dysfunction in Progranulin Deficiency

**DOI:** 10.1016/j.celrep.2017.08.056

**Published:** 2017-09-12

**Authors:** Bret M. Evers, Carlos Rodriguez-Navas, Rachel J. Tesla, Janine Prange-Kiel, Catherine R. Wasser, Kyoung Shin Yoo, Jeffrey McDonald, Basar Cenik, Thomas A. Ravenscroft, Florian Plattner, Rosa Rademakers, Gang Yu, Charles L. White, Joachim Herz

**Affiliations:** 1 Center for Translational Neurodegeneration Research, University of Texas Southwestern Medical Center, Dallas, TX 75390, USA; 2 Department of Pathology, University of Texas Southwestern Medical Center, Dallas, TX 75390, USA; 3 Department of Molecular Genetics, University of Texas Southwestern Medical Center, Dallas, TX 75390, USA; 4 Department of Cell Biology, University of Texas Southwestern Medical Center, Dallas, TX 75390, USA; 5 Department of Psychiatry, University of Texas Southwestern Medical Center, Dallas, TX 75390, USA; 6 Department of Neuroscience, University of Texas Southwestern Medical Center, Dallas, TX 75390, USA; 7 Department of Neurology and Neurotherapeutics, University of Texas Southwestern Medical Center, Dallas, TX 75390, USA; 8 Center for Human Nutrition, University of Texas Southwestern Medical Center, Dallas, TX 75390, USA; 9 Department of Neuroscience, Mayo Clinic, Jacksonville, FL 32224, USA

## Abstract

Defective lysosomal function defines many neurodegenerative diseases, such as neuronal ceroid lipofuscinoses (NCL) and Niemann-Pick type C (NPC), and is implicated in Alzheimer's disease (AD) and frontotemporal lobar degeneration (FTLD-TDP) with progranulin (PGRN) deficiency. Here, we show that PGRN is involved in lysosomal homeostasis and lipid metabolism. PGRN deficiency alters lysosome abundance and morphology in mouse neurons. Using an unbiased lipidomic approach, we found that brain lipid composition in humans and mice with PGRN deficiency shows disease-specific differences that distinguish them from normal and other pathologic groups. PGRN loss leads to an accumulation of polyunsaturated triacylglycerides, as well as a reduction of diacylglycerides and phosphatidylserines in fibroblast and enriched lysosome lipidomes. Transcriptomic analysis of PGRN-deficient mouse brains revealed distinct expression patterns of lysosomal, immune-related, and lipid metabolic genes. These findings have implications for the pathogenesis of FTLD-TDP due to PGRN deficiency and suggest lysosomal dysfunction as an underlying mechanism.

## Graphical abstract

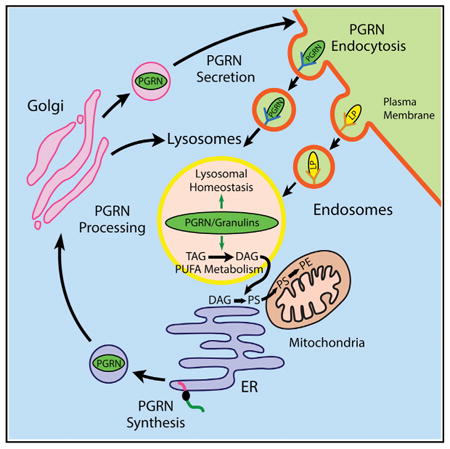

## Introduction

Lysosomes are central to lipid homeostasis via clearance of extracellular lipoproteins and autophagy. Upon fusion with lysosomes, the contents of autophagic and endocytic vesicles are catabolized by lysosomal hydrolases, and the breakdown products are released for re-use (Cuervo and Wong, 2014; Elrick et al., 2012; Maxfield, 2014). Inherited defects in lysosomal hydrolases and other lysosomal proteins lead to the accumulation of lipids and other cellular constituents within dysfunctional lysosomes. These rare inborn errors of metabolism have been broadly grouped as lysosomal storage disorders (LSDs) (Anderson et al., 2013; Kyttälä et al., 2006; Sandhoff, 2013; Schulze and Sandhoff, 2014). In comparison to other cell types, neurons are disproportionately affected in many of the prototypical LSDs, including neuronal ceroid lipofuscinose (NCL), Niemann-Pick type C (NPC), and Sandhoff disease.

Lysosomes are implicated in the pathogenesis of adult neurodegenerative diseases. In human subjects with Alzheimer's disease (AD), lysosomal dysfunction may underlie changes in brain lipid metabolism, particularly in diacylglycerides (DAGs) and phosphatidylcholines (PCs) (Bennett et al., 2013; Wood et al., 2015). Mutations in *granulin* (*GRN*), the gene encoding progranulin (PGRN), causes frontotemporal lobar degeneration (FTLD) (Cenik et al., 2012; Götzl et al., 2014, 2016; Lui et al., 2016; Nguyen et al., 2013b). The 88-kDa propeptide PGRN is trafficked to the lysosome through direct interaction with sortilin or in complex with prosaposin (PSAP), an established regulator of lysosomal lipid metabolism, via sortilin-independent mechanisms (Cenik et al., 2012; Fujita et al., 1996; Hu et al., 2010; Nguyen et al., 2013a; Nicholson et al., 2016; Zhou et al., 2015). PGRN is thought to be proteolytically processed within the lysosome into smaller subunits, called granulins, which have been implicated in the regulation of cell growth and embryonic development (Cenik et al., 2012; Nguyen et al., 2013b). However, the precise role of PGRN and granulins within the lysosome is unknown.

FTLD causes a variety of changes in behavior and in language production and comprehension (Dickson et al., 2011). The neuropathological features of FTLD include selective atrophy of the frontal and temporal lobes due to neuronal loss. At the cellular level, FTLD can be divided into subtypes based upon the immunoreactivity of characteristic subcellular protein aggregates, most commonly either transactive response (TAR) DNA-binding protein 43 (TDP-43) (FTLD-TDP) or the microtubule-associated protein tau (FTLD-tau), within neurons and/or glia (Cruts et al., 2006; Cruts and Van Broeckhoven, 2008; Dickson et al., 2011; Kleinberger et al., 2016; Pirici et al., 2006). Most FTLD cases are sporadic: ∼40% of cases are familial and usually demonstrate autosomal-dominant inheritance (Goldman et al., 2005; Götzl et al., 2016; Rosso et al., 2003). A number of genes, such as *C9orf72, MAPT* (encoding tau), and *GRN*, have been associated with these familial forms of FTLD (Baker et al., 2006; DeJesus-Hernandez et al., 2011; Neumann et al., 2006; Rademakers et al., 2012; van Swieten and Spillantini, 2007).

Nearly all human patients with FTLD due to PGRN deficiency harbor heterozygous loss-of-function (LoF) *GRN* mutations and demonstrate FTLD-TDP neuropathology (Baker et al., 2006; Cruts et al., 2006; Cruts and Van Broeckhoven, 2008). Human subjects with heterozygous LoF *GRN* mutations have elevated lysosomal proteins, including saposin D, cathepsin D, and LAMP1, within brain tissue (Götzl et al., 2014). Brain tissue from *Grn* knockout (*Grn* ^−/−^) mice exhibited increased levels of lipofuscin particles, phosphorylated TDP-43 protein, and lysosomal proteins, such as cathepsin D and LAMP1 (Ahmed et al., 2010; Filiano et al., 2013; Götzl et al., 2016; Tanaka et al., 2013, 2014; Wils et al., 2012). Patients with homozygous LoF-*GRN* mutations (Smith et al., 2012) exhibit symptoms of adultonset NCL: skin biopsies revealed enlarged lysosomes containing lamellar, pseudomembranous “fingerprint”-like inclusions of the type seen in NCL. Therefore, the pathogenesis of FTLD-TDP and NCL caused by PGRN deficiency may share gene-dosage-dependent mechanism involving lysosomal dysfunction.

We hypothesized that PGRN is involved in lysosomal homeostasis and that it participates in the metabolism of membrane lipids within the lysosome. Here, we show that PGRN deficiency alters lysosome morphology and abundance in hippocampal neurons. Using an unbiased lipidomic analysis, we show that the lipidomic profiles of human subjects and mouse models with PGRN deficiency exhibit characteristic differences in brain lipids that are gene dosage and disease specific. Loss of PGRN leads to the accumulation of long, polyunsaturated triacylglycerides (TAGs) and a concomitant reduction of DAGs and phosphatidylserines (PSs) in both the whole-cell and enriched lysosome lipidomes. Comparison between gene expression arrays from brain tissue from NPC1- and PGRN-deficient mice reveals distinct, but partially overlapping, expression patterns of lysosomal, immune-related, and lipid metabolic genes. Thus, neurodegenerative diseases caused by PGRN deficiency may resemble classical LSDs like NPC and Sandhoff disease.

## Results

### Loss of PGRN Alters Lysosomal Morphology and Abundance

Human patients and mice with homozygous LoF-*GRN* mutations develop NCL with characteristic lysosomal morphology and pseudomembranous lamellar inclusions (Smith et al., 2012). Moreover, human FTLD-TDP patients with *GRN* mutations (Götzl et al., 2014) and *Grn* knockout (*Grn*^−/−^) mice (Ahmed et al., 2010; Filiano et al., 2013; Tanaka et al., 2014; Wils et al., 2012) exhibit increased lipofuscin and immunoreactivity of lysosomal markers, such as cathepsin D and LAMP1, in brain tissue, further implicating disruption of lysosomal homeostasis with PGRN deficiency.

**Figure 1:**
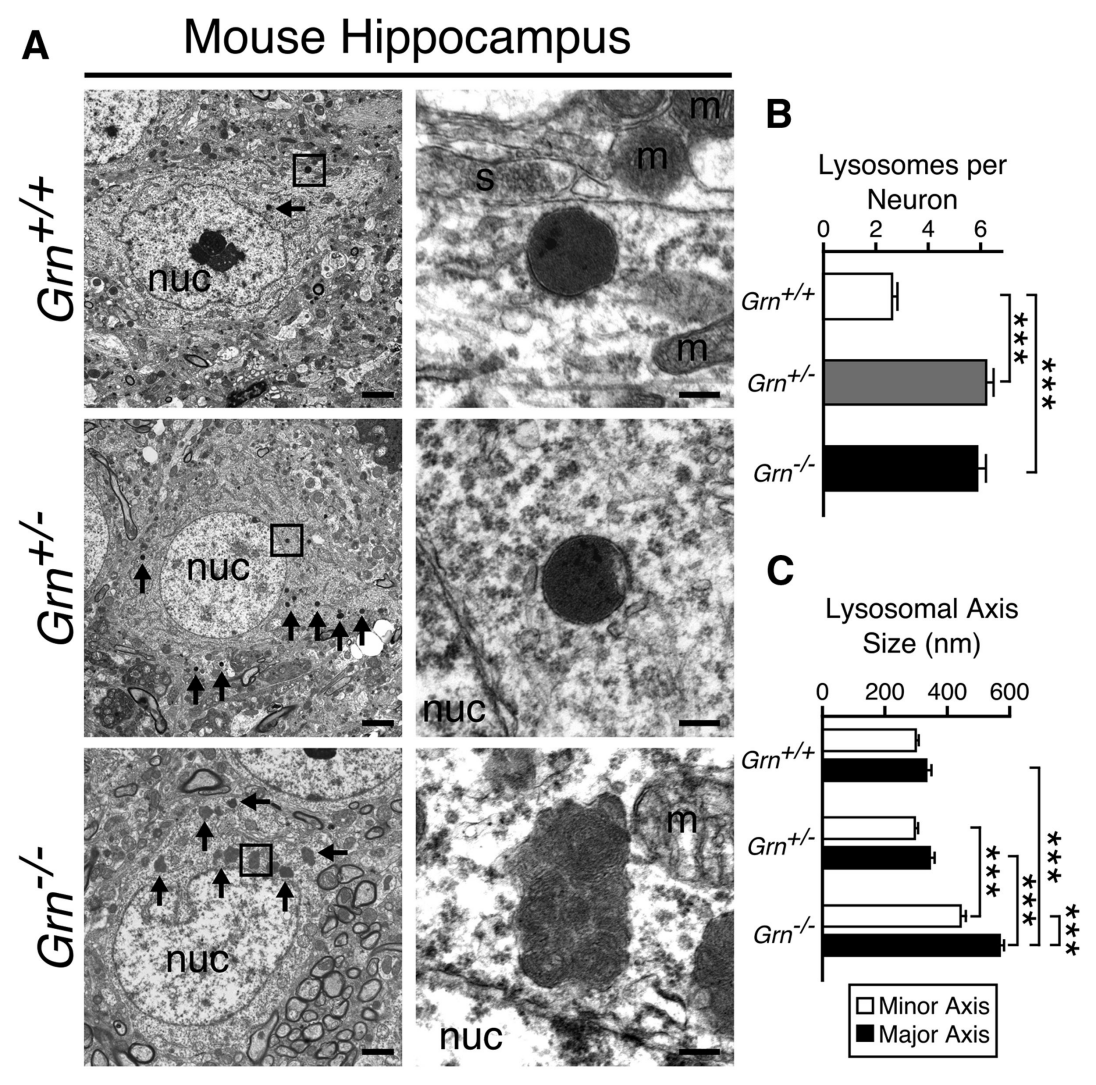
Loss of PGRN Induces Abnormal Lysosomal Morphology and Abundance in Mouse Neurons (A) Representative electron micrographs of hippocampal sections from 3-month-old *Grn*^+/+^, *Grn*^+/−^, and *Grn*^−/−^ mice. Lysosomes (inset boxes in left panels) are shown at higher magnification in right panels. Arrows indicate lysosomes. m, mitochondrion; nuc, nucleus; s, synapse. Scale bars represent 2 μm (left panels) and 200 nm (right panels). (B and C) Analysis of hippocampal lysosomes from *Grn*^+/+^, *Grn*^+/−^ and *Grn*^−/−^ mice (n = 3 mice per genotype; 20 neurons per mouse; two-tailed Student's t test; ***p < 0.001). Data are presented as mean ± SEM. (B) Quantification of lysosomes per hippocampal neuron is shown. (C) Quantification of lysosomal morphology from (B) as measured by the longest (major) and shortest (minor) axes is shown.

We used electron microscopy (Figures 1A–1C) to examine our *Grn* mutant mouse model for differences in lysosomal abundance and morphology. The shape of the lysosomes and their contents were altered in *Grn*^−/−^ mouse brains, with neuronal lysosomes exhibiting the classic fingerprint inclusions of NCL (Figures 1A and 1C). These enlarged, inclusion-filled lysosomes were ultrastructurally distinct from lipofuscin particles, which were excluded in the quantification studies (Figures 1B and 1C). Whereas there was little discernible difference in the appearance and size (Figures 1A and 1C) of neuronal lysosomes in *Grn*^+/+^ and *Grn*^+/−^ mouse brains, the total number of lysosomes was increased in neurons from *Grn*^+/−^ and *Grn*^−/−^ mice (Figure 1B).

### Lipidomic Analysis of Human and Mouse Brain Tissue

**Figure 2:**
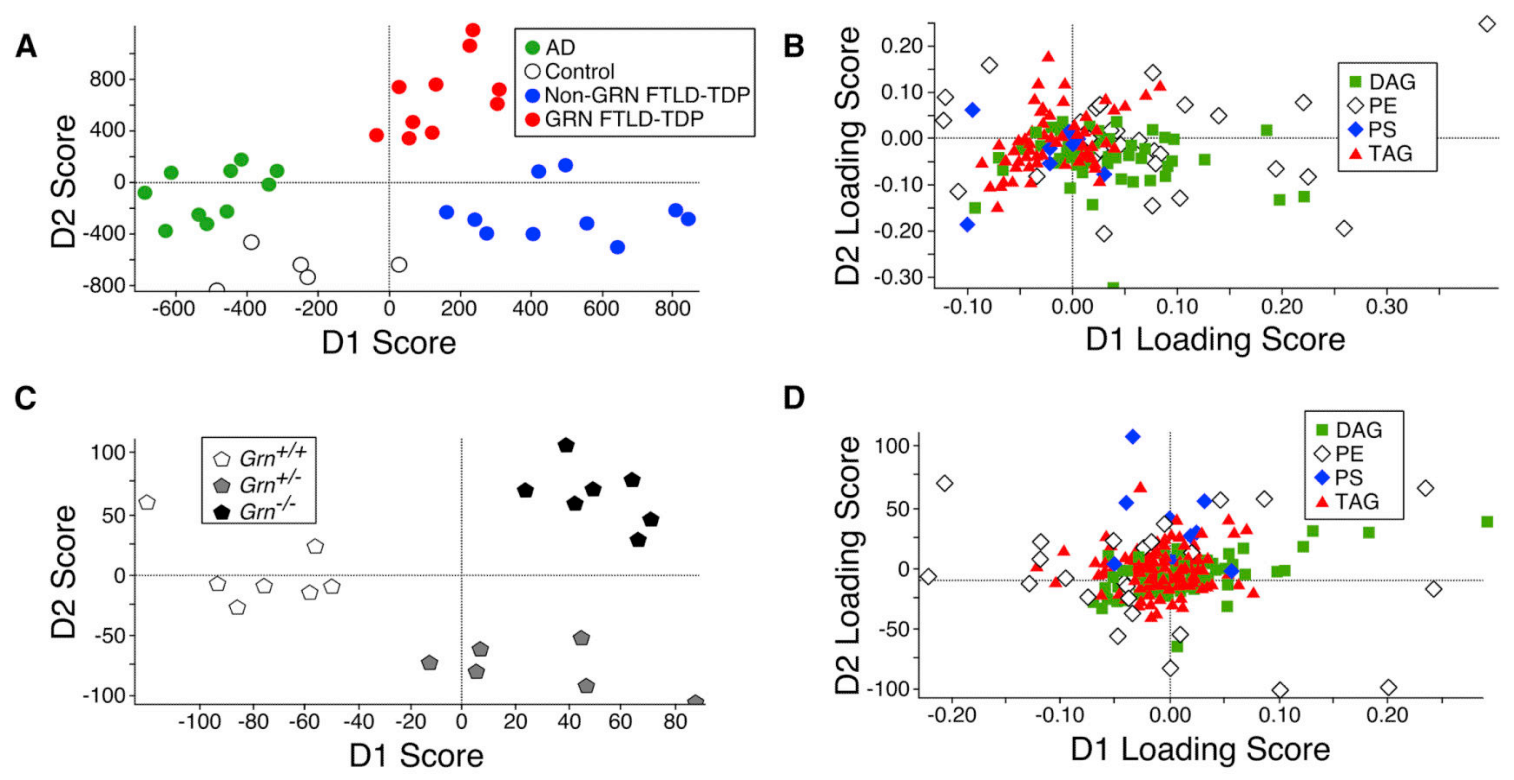
Linear Discriminant Analysis of Lipids Isolated from Pathologic Human Brains and *Grn* Mutant Mouse Brains (A and B) Fresh frozen post-mortem human cortical tissue from autopsy-confirmed AD, FTLD-TDP with and without heterozygous loss-of-function *GRN* mutations (GRN FTLD-TDP and non-GRN FTLD-TDP, respectively), and controls (n = 5; Table S1). Lipids were analyzed by infusion-based mass spectrometry (Table S2A). (A) Linear discriminant analysis (LDA) score plots using intensities and mass features of all detected lipids are shown (Table S2B). (B) LDA loading plots of TAGs, DAGs, PSs, and PEs as individual lipid classes in data from human brain samples are shown (Table S2C). (C and D) Fresh frontal cortical tissue from 12-month-old *Grn*^+/+^, *Grn*^+/−^, and *Grn*^−/−^ mice (n = 6–7). Lipids were analyzed by infusion mass spectrometry (Table S2D). (C) LDA score plots using intensities and mass features of all detected lipids are shown (Table S2E). (D) LDA loading plots of TAGs, DAGs, PSs, and PEs as individual lipid classes in data from mouse brain samples are shown (Table S2F).

The prominent lamellar lysosomal inclusions in human *GRN*^−/−^ skin fibroblasts (Smith et al., 2012) and in mouse *Grn*^−/−^ neurons (Figure 1A) are suggestive of a defect in lysosomal metabolism leading to the pathologic accumulation and storage of at least partially membrane-derived lipids (Ahmed et al., 2010; Götzl et al., 2014; Tanaka et al., 2013; Wils et al., 2012). We therefore performed an unbiased lipidomic analysis of brain tissue from human subjects with autopsy-confirmed and genetically defined neurodegenerative pathologies (Table S1; Figures 2A and 2B) and from 12-month-old *Grn* mutant mice (Figures 2C and 2D). Infusion mass spectrometry (MS/MS^ALL^) yielded 8,602 (human) and 13,214 (mouse) identified and unidentified lipid species based exclusively on mass features (Tables S2A and S2D). Linear discriminant analysis (LDA) of all 8,602 detected mass features revealed distinct changes in lipid composition in human brain lipid extracts (Figures 2A and 2B; Table S2B). Intriguingly, these changes were characteristic for different neuropathologies and allowed for discrimination between AD, FTLD-TDP due to PGRN haploinsufficiency, and non-PGRN-related forms of FTLD-TDP, based on lipid composition alone (Figure 2A). The differences between the neuropathologies were mainly driven by TAG, DAG, phosphatidylethanolamine (PE), and PS subspecies (Figure S1A; Table S2C). Similar to the human brain lipidomic analysis, a LDA score plot using all 13,214 detected mass features from mouse brain lipid extracts demonstrated distinct, genotype-specific grouping between wild-type and *Grn* mutants (Figures 2C and 2D; Table S2E). The differences between mouse genotypes were again mainly driven by TAG, DAG, PE, and PS subspecies (Figure S1B; Table S2F).

Taken together, these data suggest that a dysfunction in lysosomal lipid metabolism may underlie the characteristic and disease-specific changes of brain lipid composition in PGRN-haploinsufficient human subjects and that either PGRN or individual granulins are involved in the metabolism of these lipids.

### Lipidomic Analysis of Wild-Type and *Grn* Mutant Mouse Embryonic Fibroblasts

**Figure 3:**
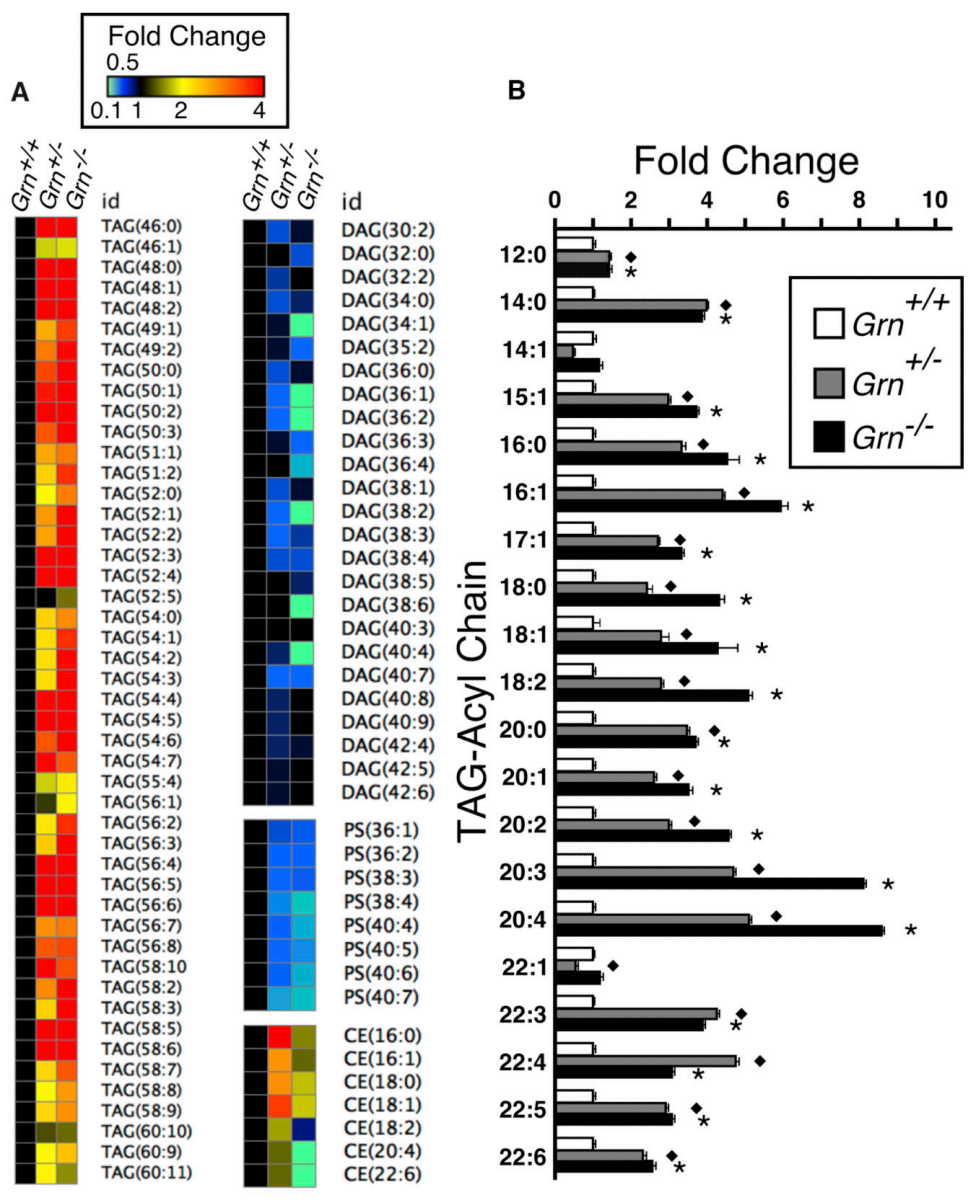
Relative Changes in Individual Lipid Species between Wild-Type and *Grn* Mutant MEFs (A) Heatmap of individual species of TAG, DAG, PS, and CE in *Grn*^+/+^, *Grn*^+/−^, and *Grn*^−/−^ MEFs (n = 3; Table S2G). Data are normalized to *Grn*^+/+^ values. (B) Plot of fold changes in select TAGs of various acyl chain lengths and unsaturation levels in *Grn*^+/+^, *Grn*^+/−^, and *Grn*^−/−^ MEFs (n = 3). Data are presented as mean ± SEM; two-tailed Student's t test; p < 0.05 between *Grn*^+/+^ and *Grn*^+/−^ (diamond) or *Grn*^−/−^ (asterisk). Data are normalized to *Grn*^+/+^ values.

Because human (Smith et al., 2012) and mouse (Figures 1A-1C) PGRN-deficient cells showed abnormal lysosomal morphology and abundance, we determined whether the characteristic differences in the lipidomic patterns seen in PGRN-haploinsufficient human and *Grn* mutant mouse brains (Figures 2 and S1) could also be observed in peripheral tissues. Total cellular lipid extracts from immortalized, non-clonal *Grn*^+/+^, *Grn*^+/−^ and *Grn*^−/−^ mouse embryonic fibroblasts (MEFs) were analyzed using our infusion-based, lipidomic mass spectrometry system. 11,642 mass features were detected, and 392 were positively identified (Table S2G). With decreasing *Grn* gene dosage, TAGs progressively accumulated and both DAGs and PSs were reduced in *Grn*^+/−^ and *Grn*^−/−^ MEFs as compared to wild-type MEFs (Figure 3A). When individual TAG species were plotted against increasing acyl chain length and unsaturation level, PGRN deficiency led to a preferential accumulation of TAGs with long acyl chains and high unsaturation levels (ranging to more than an 8-fold increase in some TAG species) in *Grn*^+/−^ and *Grn*^−/−^ MEFs as compared to wild-type MEFs (Figure 3B). Cholesteryl esters (CEs) also exhibited a distinct genotype-specific pattern, with a relative increase in CEs containing saturated and monounsaturated acyl chains in *Grn*^+/−^ and a concomitant reduction in CEs containing polyunsaturated acyl chains in *Grn*^−/−^ cells, potentially arising from reduced substrate availability to acyl-coenzyme A (CoA):cholesterol acyltransferase caused by the complete loss of PGRN and granulin function.

These data suggest that PGRN or individual granulins participate in the metabolism of long-chain, polyunsaturated fatty acids, possibly by influencing the activity of lysosomal metabolic enzymes. This may involve the conversion of other lipid species, such as phosphatidic acid, to DAGs or the egress of DAGs from the lysosometo other compartments, i.e., the endoplasmic reticulum (ER) (Flis and Daum, 2013; Helle et al., 2013; Lev, 2010). The lipid profile changes observed in MEFs are consistent with the data obtained from the human and mouse brain samples, further supporting our hypothesis that PGRN participates in lipid metabolism within the cell, presumably within the lysosome itself.

### Lipidomic Analysis of Lysosome-Enriched Liver Organelles

The liver is the central clearing house for lipids in the body, where nutritional lipids are first received and then repackaged for redistribution to peripheral tissues, mainly muscle and adipose tissue (Nguyen et al., 2008). It is also the site of the highest peripheral PGRN expression, and cell fractionation procedures for the selective enrichment of biochemical quantities of hepatocyte organelles, including lysosomes, have been well established. Using a standard protocol (Graham, 2001), we generated subcellular fractions highly enriched for lysosomes (Figures S2A–S2C). Fractionation efficiency was tested using a variety of organelle markers for lysosomes (NPC1, LAMP1, and cathepsin D), endosomes (EEA1), plasma membrane (Na^+^/K^+^ ATPase), ER (ACAT1), Golgi (Gm130), and peroxisomes (PMP70; Figure S2D). Unprocessed PGRN did not appear to be enriched in lysosomes compared to whole-cell lysate (Figure S2E). This may be due to PGRN precursor sequestration within secretory vesicles and not in lysosomesorrapid processing of the precursor to granulins within lysosomes.

**Figure 4:**
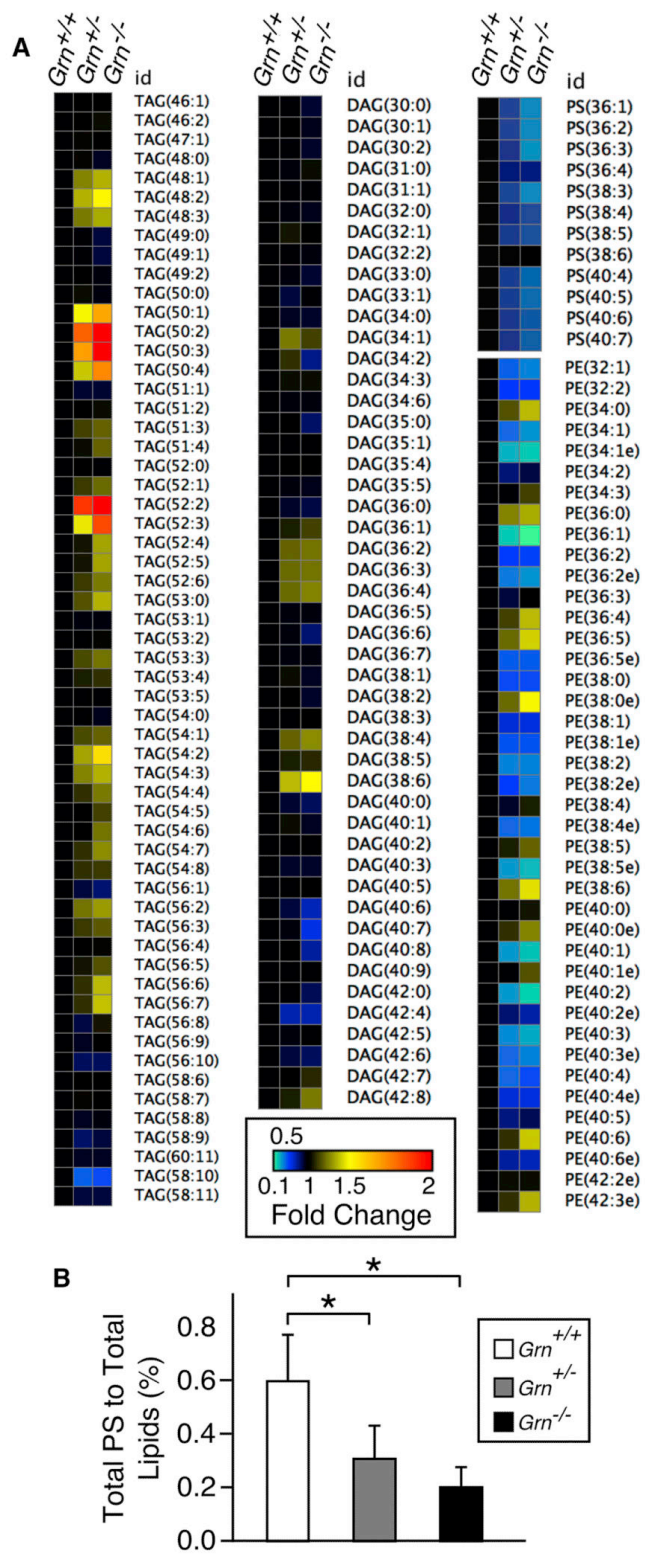
Relative Changes in Individual Lipid Species from Enriched Hepatic Lysosome Fractions between Wild-Type and *Grn* Mutant Mice (A and B) Lysosomes were enriched from livers of 4-month-old *Grn*^+/+^, *Grn*^+/−^ and *Grn*^−/−^ mice (n = 3 per genotype), and the extracted lipids were analyzed (TableS2H). (A) Heatmap of individual species of TAGs, DAGs, PSs, and PEs in enriched lysosomes is shown. Data are normalized to *Grn*^+/+^ values. (B) Total PS levels relative to total lipid content (%) from enriched hepatic lysosomes isolated in (A) are shown. Data are presented as mean ± SEM; two-tailed Student's t test; *p < 0.05.

Infusion-based lipidomics of the enriched lysosomes (Figure 4; Table S2H) revealed a similar gene-dosage-dependent increase in a subset of TAGs with a pronounced reduction in the majority of PSs and PEs (Figures 4A and 4B) but no significant change in DAGs (Figure 4A). The differences between the MEF and liver lysosome lipidomes may be attributed to physiologic differences between cultured MEF cells and hepatic tissue. For example, MEFs endogenously synthesize virtually all lipids, whereas hepatocytes and other liver cells receive a significant portion of their lipids exogenously from the diet.

Various LSDs are due to a deficiency of specific lysosomal hydrolases (Kyttälä et al., 2006; Schulze and Sandhoff, 2014). Though not a hydrolase itself, NPC1 can affect the functioning of lysosomal hydrolases and overall lysosomal activation, as measured by cathepsin B activity, through the aberrant accumulation of lipids within the lysosome (Elrick et al., 2012). Lysosome enrichment by subcellular fractionation showed, for example, that the complete absence of PGRN in lysosomes increased cathepsin D expression and altered its processing (Figure S3).

### Transcriptomic Analysis of PRGN-Deficient Mouse Brains

**Figure 5:**
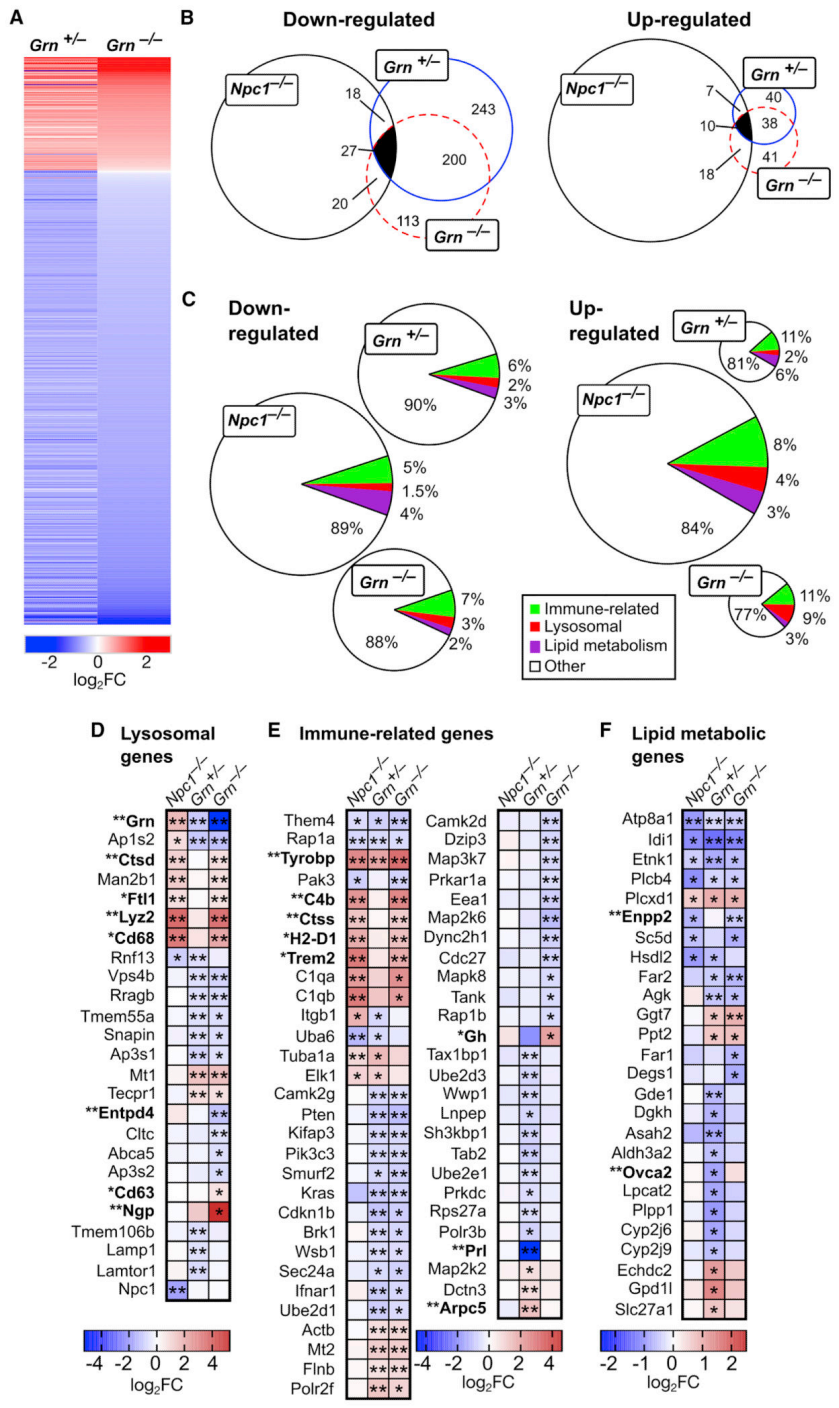
Transcriptomic Analysis of *Grn*^+/−^, *Grn*^+/−^, and *Np*c1^−/−^ Mutant Mouse Brains (A–C) Sequencing of total mRNA extracted from whole brains from 7−month−old female *Grn*^+/+^, *Grn*^+/−^, and *Grn*^−/−^ mice (n = 2). All transcripts with > 1.5-fold change (p < 0.05) were considered differentially expressed. (A) Heatmap of the log_2_ fold change of all genes differentially expressed in *Grn*^+/−^ and *Grn*^−/−^ brains as compared to *Grn*^+/+^ brains is shown (Table S3). (B) Proportional Venn diagram illustrating the overlap of down- (left) and upregulated (right) transcripts in *Grn*^+/−^ and *Grn*^+/−^ brains with previously published *Npc1*^−/−^ microarray data is shown (Alam et al., 2012; Tables S4 and S5). Numbers of shared genes are indicated. (C) Pie charts depicting the proportion of genes associated with lysosomal function, immune response, and lipid metabolism altered in relation to all up- and downregulated transcripts in (B) are shown (Tables S4 and S5). (D-F) Comparison of gene expression between brains from *Grn*^+/−^, *Grn*^−/−^, *Npc1*^−/−^, and *Grn*^+/+^ mice. The gene sets were limited to genes significantly changed in brains from either *Grn*^+/−^ or *Grn*^+/−^ mice as compared to *Grn*^+/+^ controls. Asterisks indicate significant changes (*q < 0.05; **q < 0.01). Genes differentially expressed in *Grn*^+/−^ brains as compared to *Grn*^+/−^ brains are bolded. Genes associated with (D) lysosomal function, (E) immune response, and (F) lipid metabolism are indicated.

A recent study using microarray profiling showed increased expression of lysosomal and innate immunity genes in the brains of *Grn*^−/−^ mice (Lui et al., 2016). To obtain a complete overview of the entire complement of RNA expression that is altered in *Grn* deficiency, with a particular focus on transcripts involved in lipid metabolism, we sequenced the mRNA extracted from the brains of 7-month-old *Grn*^+/+^, *Grn*^+/−^, and *Grn*^−/−^ mice. Of 22,552 known mouse genes, loss of one or both alleles of *Grn* altered the transcription of 774 genes compared to *Grn*^+/+^ controls (Figure 5A; Table S3). Of the transcripts differentially expressed in either *Grn* ^+ −^ or *Grn*^−/−^ brains, approximately 50% were similarly and significantly altered in both genotypes (Figure 5B; Tables S4 and S5). Several differentially expressed transcripts in both *Grn*^+/−^ and *Grn*^−/−^ mice have functions in lipid metabolism (Figure 5C; Tables S4 and S5). Of these, a subset was shared between *Npc1*^−/−^ and *Grn* mutants. Moreover, brain mRNA levels of several lipid hydrolases with functions consistent with compensatory changes to the cellular lipidome were significantly altered in *Grn*^+/−^ and *Grn*^−/−^ mice as compared to *Grn*^+/+^ controls (Figure S4). Our transcriptomic analysis of mRNAs from *Grn* mutant mouse brains was independently validated by the concordant results of innate immunity genes that matched those previously reported by Lui and colleagues using a microarray approach (Lui et al., 2016).

To determine whether PGRN deficiency results in similar transcriptional changes as seen in other mouse models of lysosomal storage diseases, we used a publicly available microarray dataset of brain mRNAs isolated from *Npc1*-deficient (*Npc1*^−/−^ mice (Alam et al., 2012). Significantly altered transcripts were compared between the transcriptomic datasets from *Npc1*^−/−^ *Grn*^+/−^ and *Grn*^−/−^ mice (Figures 5B and 5C; Tables S4 and S5). Of the differentially regulated genes in *Grn*^+/−^ and *Grn*^−/−^ brains, a fraction was similarly up-or downregulated in *Npc1*^−/−^ brains. A subset of the shared upregulated genes in the *Npc1*^−/−^ and *Grn* mutant brains (Figure 5B) are associated with either lysosomal function or the immune response (Table S5). These findings are consistent with the results in *Npc1*-deficient mouse brains where immune-related and lysosomal genes are upregulated (Alam et al., 2012).

The magnitude of change among lysosomal, immune-related, and lipid metabolic genes that were differentially regulated in *Grn*^+/−^ and *Grn*^−/−^ brains was *Grn* dose dependent (Figures 5D–5F). Importantly, the transcriptomic data revealed distinct, but partially overlapping, expression patterns (Figures 5D-5F). This observation is consistent with the notion that pathophysiological molecular mechanisms are, in part, differentially controlled by NPC1 and PGRN (Cenik et al., 2012; Götzl et al., 2014, 2016; Kwon et al., 2009; Nicholson et al., 2016).

These data suggest that a reduction or complete loss of PGRN induces characteristic changes in the expression of genes associated with lysosomal function, the immune response, and lipid metabolism, indicative of lysosomal dysfunction. These findings further indicate that neurodegenerative diseases due to PGRN deficiency may be driven by similar pathophysiologic mechanisms as classic LSDs, such as NPC disease.

## Discussion

PGRN deficiency manifests itself clinically as two phenotypically distinct diseases, depending on whether the loss of one functional *GRN* allele induces a state of haploinsufficiency or complete loss of function when both alleles are defective. In the first case, PGRN haploinsufficiency results in a dominant form of FTLD-TDP, in the latter a recessive form of NCL. An explanation for these dramatically different manifestations of the same gene defect likely resides in the lysosomal function of PGRN and the granulins that are hypothesized to be proteolytic cleavage products of PGRN. A fraction of newly synthesized PGRN travels through the secretory pathway in a complex with PSAP, another propeptide that undergoes cleavage to mature saposins (Kishimoto et al., 1992; Nicholson et al., 2016; Zhou et al., 2015). In contrast to PGRN and its granulins, the role of saposins as cofactors for lysosomal lipid hydrolases is well established (Schulze and Sandhoff, 2014; Sun et al., 2013; Vaccaro et al., 2010). We hypothesized that PGRN and the granulins might have similar functions in the lysosome. This hypothesis was supported by the striking lysosomal storage phenotype that occurs in the complete absence of PGRN and by the characteristic lysosomal inclusions of lamellar pseudomembranous structures (Figure 1), which are indicative of an inability of the lysosome to digest and recycle cellular membranes.

Decreasing lysosomal capacity is now being recognized as a characteristic feature of the aging process (Rubinsztein et al., 2011), which is also the greatest risk factor for dementias, such as AD and FTLD. Hence, we hypothesized that the clinical manifestations of FTLD-TDP in humans might be indicative of an unmasking of a latent lysosomal deficiency caused by PGRN haploinsufficiency. Characteristic changes in the brain lipidome have been previously reported for AD (Bennett et al., 2013; Wood et al., 2015). Analysis of brain lipids extracted from age-matched and autopsy-confirmed control, AD, non-GRN FTLD-TDP, and GRN FTLD-TDP human subjects reproduced and extended these findings (Figures 2 and S1). Intriguingly, using LDA as an analytical tool, none of the distinct pathologic or control lipidomes overlapped with each other, indicating that normal aging and different age-related neurodegenerative disorders differentially affect cellular lipid metabolism and homeostasis. More specifically, the lipid changes seen in GRN FTLD-TDP human subjects closely resembled the lipidome of *Grn*^+/−^ and *Grn*^−/−^ mice, with preferential changes in TAGs, DAGs, and PSs (Figures 2 and S1). This was largely recapitulated in the whole-cell lipidome from *Grn* mutant MEFs, with a preferential, gene-dosage-dependent accumulation of CEs and TAGs and a corresponding decrease of DAGs and PSs of increasing chain length and unsaturated bonds (Figure 3). Similar results were obtained when we analyzed lysosomes enriched from wild-type and *Grn* mutant mouse livers (Figure 4A), which further revealed a striking approximate 3-fold decrease in the PS/total lipid ratio (Figure 4B). In addition, *Grn*^−/−^ ver lysosomes also showed a significant increase in both unprocessed and processed cathepsin D, consistent with a compensatory response of the lysosome to a PGRN or granulin-deficiency-induced malfunction (Figure S3). Taken together, these findings indicate a PGRN-or granulin-dependent mechanism in the transfer of long, unsaturated fatty acids from CEs and TAGs to PSs and PEs and to a potential role in the function of cathepsins. Homozygous deficiencies of cathepsin D and cathepsin F independently cause their own genetically distinct forms of NCL, indicating that normal functioning of lysosomal hydrolases is essential for preventing NCL-type storage diseases (Anderson et al., 2013; Koike et al., 2000; Kyttälä et al., 2006; Stoka et al., 2016).

Our unbiased comprehensive lipidomic analysis was complemented by a transcriptomic survey of mRNA extracted from *Grn*^+/+^, *Grn*^+/−^ and *Grn*^−/−^ mouse brains. Analysis of the changes in lysosomal and immune-related gene expression by RNA sequencing (RNA-seq) (Figure 5) yielded results that reproduced those previously published (Lui et al., 2016) using a technically different microarray-based approach. Moreover, our RNA-seq data allowed us to quantitatively compare transcriptomic changes in *Grn* deficiency with a previously reported dataset obtained in *Npc1* knockout (*Npc1*^−/−^) mouse brains (Figures 5B–5F), revealing selective changes in the expression of lysosomal, immune-related, and lipid-related genes between NPC1 and PGRN deficiency states. Despite a state of severe lysosomal dysfunction that is common between *Npc1* and *Grn* mutant mice, there was a highly selective subset of transcripts in all three categories that were significantly altered in *Grn*^−/−^ mice, but not in *Npc1*^−/−^ mice (Figures 5D-5F). These selective differences point toward pathology-specific mechanisms by which dysfunctional lysosomes communicate with the cellular gene expression machinery. A possible mechanism might involve the differential complement of cellular lipids that accumulate as a result of the different biochemical defects, leading to the activation or suppression of lipid-sensitive nuclear hormone receptors that regulate the transcription of numerous metabolic genes, including those involved in lipid metabolism. These include a group of lipid hydrolases that exhibited significant differential regulation in *Grn* deficiency (Figure S4), suggesting roles in metabolic pathways that depend on the function of PGRN or of individual granulins.

In summary, we present here a comprehensive set of lipidomic and transcriptomic data that support a role for PGRN and granulins in the metabolism of lipids and proteases within the lysosome. We propose that neurodegenerative diseases due to PGRN dysfunction (FTLD-TDP with PGRN haploinsufficiency) involve latent lysosomal dysfunction. This metabolic defect consequently exacerbates the progressive reduction of lysosomal and autophagic capacity during normal human aging and becomes the driver for the neuronal loss that culminates in FTLD-TDP.

## Experimental Procedures

### Generation of *Grn* Mutant Mice and Animal Husbandry

The generation of *Grn* mutant (*Grn*^+/−^ and *Grn*^−/−^) mice has been previously described (Martens et al., 2012). See Supplemental Experimental Procedures for more detail. All procedures were performed in accordance with the protocols approved by the Institutional Animal Care and Use Committee of the University of Texas Southwestern Medical Center.

### mRNA Sequencing from *Grn* Mutant Mouse Brains

Total RNA was extracted from the brains of female *Grn*^+/+^, *Grn*^+/−^, and *Grn*^−/−^ mice (n = 2; average age = 200 ± 23 days) with Trizol (Ambion). mRNA libraries were prepared from each RNA sample and sequenced via Illumina sequencer. High-quality reads were mapped to the genome and analyzed to determine differentially expressed transcripts between genotypes. See Supplemental Experimental Procedures for more detail.

### Extraction and Analysis of Lipids Samples by Infusion-Based MS/MS ALL

Frozen pieces of fresh brain tissue were isolated from *Grn*^+/+^, *Grn*^+/−^, and *Grn*^−/−^ mice and from autopsy-confirmed cases (Table S1) of AD, FTLD-TDP with and without *GRN* hemizygous loss (GRN FTLD-TDP and non-GRN FTLD-TDP, respectively), and controls with only age-related neuropathologic changes (control) and were prepared for lipidomic MS/MS^ALL^ analysis. MEFs and hepatic lysosomes were harvested and prepared for lipidomic MS/MS^ALL^ analysis. See Supplemental Experimental Procedures for more detail.

### Statistical Analysis and Reporting

The sample size, number of replicates, and statistical tests are indicated either in the text, figure legends, and/or Experimental Procedures. No data were excluded from analysis, and any and all outliers encountered within the data were included in the statistical analysis. Data generated from the transcriptomic analysis and high-throughput lipidomic analysis has been provided as supplemental tables and Excel files.

## Data and Software Availability

The accession number for the RNA-seq data reported in this paper is SRA: SRP114906.

## Supplementary Material

1

2

3

4

## Highlights

- Altered neuronal lysosome abundance and morphology in PGRN deficiency
- Neuropathologic and lysosome-specific lipidomics of PGRN deficiency
- Disruption of polyunsaturated TAGs, DAGs, and PSs in PGRN deficiency
- Distinct lysosomo-, immuno-, and lipo-transcriptomics of PGRN and NPC1 deficiency
