# Supplementary material for: Lipidomic and Transcriptomic Basis of Lysosomal Dysfunction in Progranulin Deficiency
Source: Cell Rep. Author manuscript; Available in PMC 2018 Jan 8. (PMC5757843; doi:10.1016/j.celrep.2017.08.056)
Supplement: 1 [file NIHMS902297-supplement-1.pdf]

SUPPLEMENTAL FIGURES

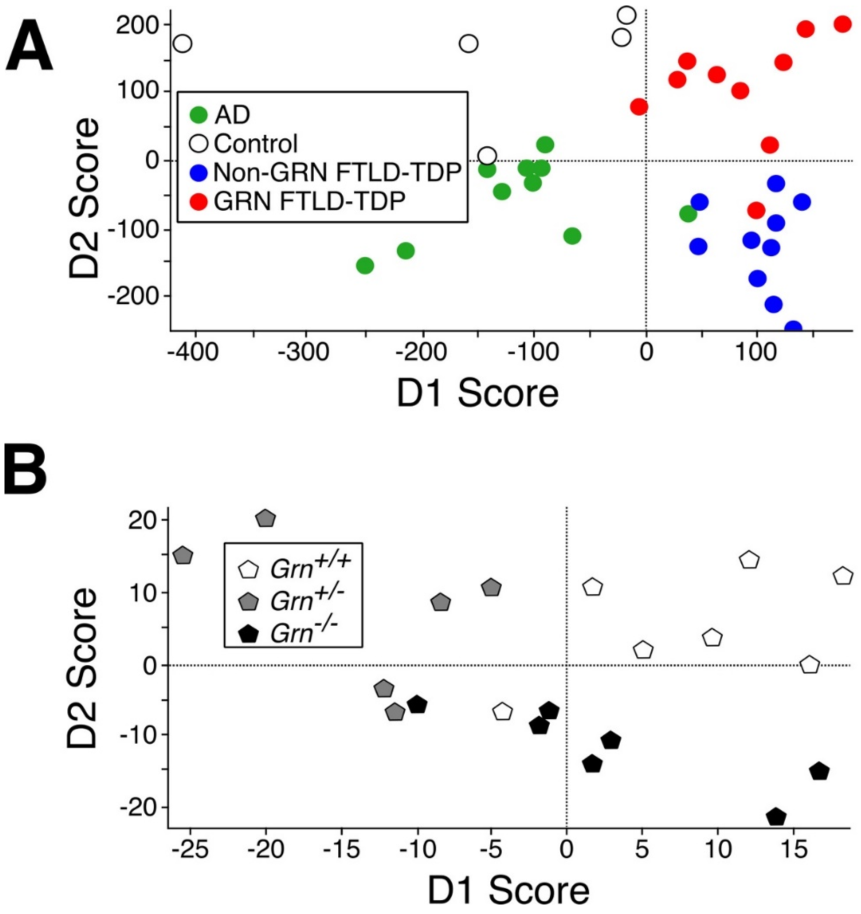

Figure S1. Linear discriminant analysis (LDA) of specific lipid subspecies isolated from pathologic human brains and *Grn* mutant mouse brains. Related to Figure 2.

(A and B) Brain tissue isolated from human subjects with various autopsy-confirmed neurodegenerative diseases (see also Table S1) and from *Grn* mutant mice were subjected to lipidomic analysis (see also Tables S2 and S4, respectively). (A) LDA score plot of brain samples from human subjects with AD, FTLD-TDP with and without heterozygous loss-of-function *GRN* mutations (GRN FTLD-TDP and Non-GRN FTLD-TDP, respectively) and controls with age-related neuropathologic changes (Control) using the intensities of TAGs, DAGs, PSs, and PEs (see also Table S3B). (B) LDA score plot of brain samples from $Grn^{+/+}$, $Grn^{+/-}$, and $Grn^{-/-}$ mice using the intensities for the same lipid classes in (A) (see also Table S5B).

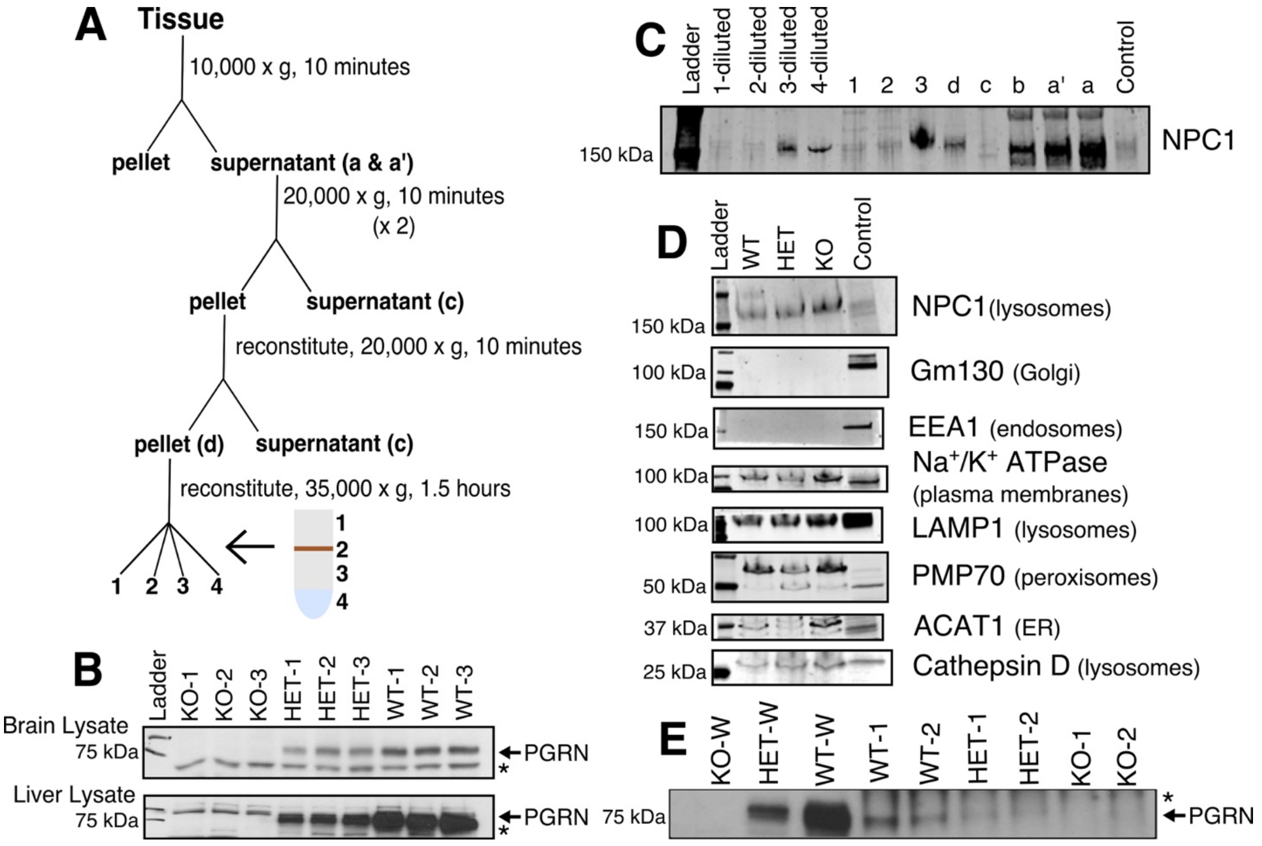

Figure S2. Lysosome enrichment from mouse livers. Related to Figure 4.

(A) Flow diagram of ultracentrifugation protocol for lysosome enrichment. Tissue was homogenized in HM buffer, centrifuged at 10,000 x g for 10 minutes, and the supernatant retained (**a**). This was repeated, and the resulting supernatant was added to the previously saved supernatant (**a'**). This supernatant was then centrifuged at 20,000 x g for 10 minutes and discarded. The resulting pellet was reconstituted in HM buffer and centrifuged at 20,000 x g for 10 minutes. The supernatant was again discarded (**c**), and the resultant pellet was then reconstituted in HM buffer. Percoll in percoll diluent was added, and the mixture was centrifuged at 35,000 x g for 1.5 hours. This ultracentrifugation step created a Percoll gradient composed of 4 fractions.

(B) PGRN immunoblots of individual whole brain and liver lysates (50 μg protein per sample) from 4-month-old $Grn^{+/+}$ [WT], $Grn^{+/-}$ [HET], and $Grn^{-/-}$ [KO] mice. Asterisks [*] denote non-specific bands.

(C) Starting with liver lysate from a 4-month-old wild type mouse, each step in (A) was probed for the lysosome marker NPC1. Fraction "3" showed strong bands for the lysosome marker while fractions "1" and "2" showed no bands. Fraction "4" consisted of a semi-solid gel which made immunoblotting difficult. Thus, fraction "3" was used to check the purity of lysosome enrichment. Whole cell lysate from $Grn^{+/+}$ MEFs was used as a control.

(D) To verify membrane purity, we probed fraction "3" from individual lysosomal preparations (liver from 4-month-old $Grn^{+/+}$ [WT], $Grn^{+/-}$ [HET], and $Grn^{-/-}$ [KO] mice) with a battery of markers specific for various subcellular compartments. Whole cell lysate from $Grn^{+/+}$ MEFs was used as a control.

(E) Immunoblotting for PGRN in individual fraction "3" liver preparations from 4-month-old $Grn^{+/+}$ [WT], $Grn^{+/-}$ [HET], and $Grn^{-/-}$ [KO] mice. Whole liver lysates (WT-W, HET-W, and KO-W) were used as controls. Asterisk [*] denotes non-specific bands.

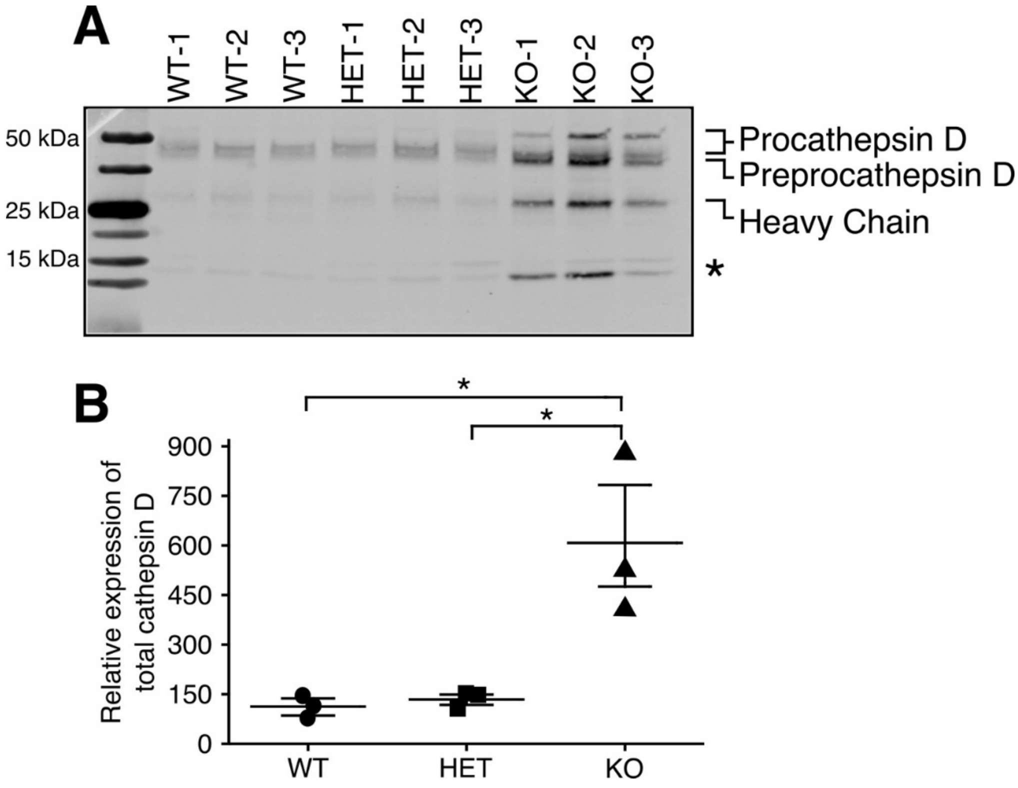

Figure S3. Cathepsin D processing in lysosome-enriched organellar fractions from wild type and *Grn* mutant mouse livers. Related to Figure 4.

(A) Cathepsin D immunoblots of lysosome-enriched subcellular organellar preparations (1.25 μg protein per lane) isolated from livers of 4-month-old $Grn^{+/+}$ (WT), $Grn^{+/-}$ (HET), and $Grn^{-/-}$ (KO) mice (n = 3 per genotype). Data presented as mean ± SEM. Asterisk [*] indicates a likely degradation product.

(B) Plot of relative expression of total cathepsin D from quantified band intensities in (A). Data presented as mean ± SEM; two-tailed Student's t-test; $*p < 0.05$.

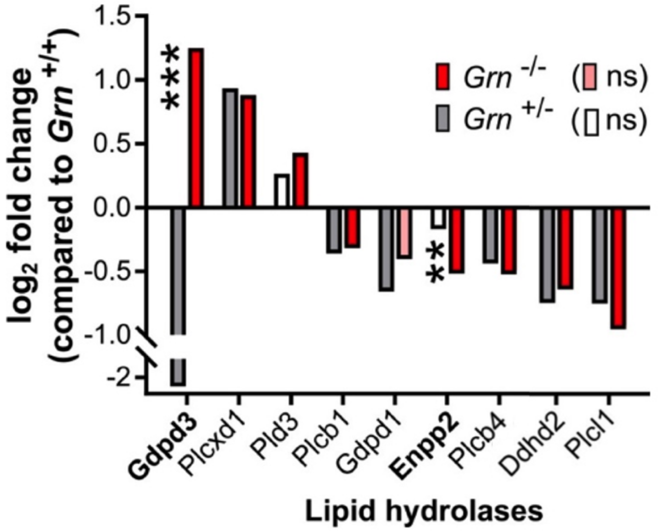

Enzyme	Substrate(s)	Product(s)
Glycerophosphodiester phosphodiesterase domain-containing protein 3 (Gdpd3) (Ohshima et al., 2015)	1-acyl-lyso-PC and lyso-PAF	Lysophosphatidic acids (LPAs) and alkyl-LPAs
Phospholipase C, phosphatidylinositol-specific, X domain-containing protein (Plcxd1) (Gellatly et al., 2012; Irvine 2016)	PIP_2	IP_3 and DAGs, other phosphoinositols
Phospholipase D member 3 (Pld3) (Chung et al., 1997; Irvine 2016; Nelson and Frohman 2015)	Membrane phospholipids, especially phosphatidylcholine	Phosphatidic acids
Phospholipase C beta-1 (Plcb1) (Irvine 2016)	PIP_2	IP_3 and DAGs
Glycerophosphodiester phosphodiesterase domain-containing protein 1 (Gdpd1) (Ohshima et al., 2015)	1-acyl-lyso-PC and lyso-PAF	Lysophosphatidic acids (LPAs) and alkyl-LPAs
Ectonucleotide pyrophosphatase/phosphodiesterase 2 (Enpp2/autotaxin) (Tokumura et al., 2002)	Lysophospholipids, especially lysophosphatidylcholine	Lysophosphatidic acids (LPAs)
Phospholipase C beta-4 (Plcb4) (Irvine 2016)	PIP_2	IP_3 and DAGs
DDHD domain-containing protein 2 (Ddhd2) (Sato et al., 2010)	sn-1 ester bonds of various phospholipids	2-acyl-lysophospholipids and fatty acids
Phospholipase C-like 1 (Plcl1) (Irvine 2016)	PIP_2	IP_3 and DAGs

Figure S4. Relative changes in lipid hydrolase transcripts in *Grn*$^{+/-}$ and *Grn*$^{-/-}$ mouse brains. Related to Figure 5. Plot depicts the $\log_2$ fold change in lipid hydrolase transcripts in *Grn*$^{+/-}$ and *Grn*$^{-/-}$ mouse brains as compared to *Grn*$^{+/+}$ brains quantified from mRNA sequencing. Lighter colored bars represent insignificant changes ($q > 0.05$). Genes differentially expressed in *Grn*$^{-/-}$ brains as compared to *Grn*$^{+/-}$ brains are bolded. Data presented as mean ± SEM. Asterisks indicate significant differences between *Grn*$^{+/-}$ and *Grn*$^{-/-}$ values (**$q < 0.01$; ***$q < 0.001$).

Table S1. Human subject demographics. Related to Figure 2.

Lipidomic group	Case	Diagnosis	Age at death	Gender	Ethnicity	*GRN* mutation
GRN FTLD-TDP	12900	FTLD-TDP, *GRN* mutant	71	M	Caucasian	p.Arg493*
	19870	FTLD-TDP, *GRN* mutant	65	M	Caucasian	p.Cys253*
	19943	FTLD-TDP, *GRN* mutant	73	F	Caucasian	p.Asp47fs
	26115	FTLD-TDP, *GRN* mutant	61	M	Caucasian	p.Arg493*
	27990	FTLD-TDP, *GRN* mutant	61	F	Caucasian	p.Arg418*
Non-GRN FTLD-TDP	16816	FTLD-TDP, *GRN* WT	76	M	Caucasian	Negative
	23029	FTLD-TDP, *GRN* WT	72	F	Caucasian	Negative
	25719	FTLD-TDP, *GRN* WT	68	M	Caucasian	Negative
	25949	FTLD-TDP, *GRN* WT	76	M	Caucasian	Negative
	31062	FTLD-TDP, *GRN* WT	57	M	Caucasian	Negative
AD	41845	AD	65	M	Caucasian	Negative
	41969	AD	82	F	Caucasian	Negative
	43045	AD	69	M	Caucasian	Negative
	43755	AD	77	M	Caucasian	Negative
	44810	AD	56	F	Caucasian	Negative
Control	42133	Control	100	F	Caucasian	Negative
	42990	Control	84	F	Caucasian	Negative
	45116	Control	78	M	Caucasian	Negative
	45329	Control	78	M	Caucasian	Negative
	46202	Control	77	M	Caucasian	Negative

Table S1. Related to Figure 2. Table of human subject demographics.

Table S5: Overlap of differentially expressed genes. Related to Figure 5.

	Genotypes	#	Genes
**Down-Regulated**	*Grn*$^{-/-}$↓, *Grn*$^{+/-}$↓	227	Rap1a, Them4, Brk1, Camk2g, Cdkn1b, Ifnar1, Kifap3, Kras, Pik3c3, Pten, Sec24a, Smurf2, Ube2d1, Wsb1, Ap1s Ap3s1, Grn, Rragb, Snapin, Tmem55a, Vps4b, Idi1, Etnk1, 3632451O06Rik, Brix1, Capza1, Ccny, Fam13a, Glr Hspa4, Jam2, Lifr, Lrp1b, Luzp2, Lztfl1, Nefh, Opalin, Phip, Plp1, Prepl, Ptpn4, Ralgapa1, Sycp1, Ttc21b, Ube2 Ublcp1, Usp9x, 1110032A03Rik, 3830406C13Rik, A830010M20Rik, Aak1, Abcg2, Adtrp, Aff4, Ankrd45, Arhgap3 Asnsd1, Atp11b, Atp11c, Atp5e, BC030336, Bhlhe41, Bod1l, Btf3l4, C1d, Cacna1e, Capn7, Caprin2, Ccdc18 Ccdc50, Ccdc88a, Cd2ap, Cdr1, Cdv3, Cenpv, Cep290, Cep70, Cfl2, Chm, Clcn3, Cmah, Cntd1, Commd3, Copb Cops4, Cox7a2l, Cse1l, Cul4b, Dbp, Dcun1d1, Ddhd2, Dlg1, Dnajc15, Dnajc2, Dpm1, Dpp10, Efr3a, Eif2a, Elmod Emc2, Epc2, Etf1, Fam135a, Fam175b, Fam92a, Fam98a, Fgfr1op2, Foxj3, Gabra2, Gatad1, Gcc2, Gcfc2, Gfm Gins4, Glo1, Gm11837, Gm17821, Gm1821, Gm3985, Gnpda2, Golga5, Gpr22, Grcc10, Hook1, Hp1bp3, Hsf Ift74, Ift81, Ireb2, Kdelc1, Kmt2e, Krt12, Lnx1, Lrch2, Ltv1, Man2a1, Map9, Mbd5, Mettl14, Mga, Mkrn2, Mppe Mtpn, Ncoa7, Ndufb4, Ndufb5, Npat, Ogfrl1, Orc3, Osbpl8, Papd4, Papolg, Pcm1, Pcnp, Pdcd4, Pdcd5, Pgm Phax, Pnpt1, Polk, Ppig, Ppp1cc, Ppp1r7, Prpf39, Prune2, Ptpn22, Ptrh2, Rab23, Rab2a, Rabep1, Ranbp2, Ranbp Rbm26, Rgs4, Rgs7, Rhot1, Rnf11, Rnf146, Rnpc3, Rpgrip1l, Rpl18a, Rpl35, Scai, Sclt1, Serp2, Sf3b6, Slc24a Slit2, Smim19, Snapc3, Snx13, Snx27, Snx4, Specc1, Spopl, Stambpl1, Stau2, Supt16, Taok1, Taok3, Tbc1d2 Tcea1, Tenm1, Tfg, Tgs1, Tmem181a, Tmem181b-ps, Tmem183a, Tomm6, Trim33, Trip11, Trmt10b, Ttc13, Ube3 Uck1, Uggt2, Upf2, Ust, Vps26a, Vps41, Wdr36, Ythdf3, Zbtb11, Zcchc7, Zfp14, Zfp280c, Zfp638
	Npc1$^{-/-}$↓, *Grn*$^{+/-}$↓	45	Rap1a, Them4, Uba6, Rnf13, Idi1, Hsdl2, 3632451O06Rik, Brix1, Capza1, Ccny, Fam13a, Glrb, Hspa4, Jam2, Li Lrp1b, Luzp2, Lztfl1, Nefh, Opalin, Phip, Plp1, Prepl, Ptpn4, Ralgapa1, Sycp1, Ttc21b, Ube2b, Ublcp1, Usp9x, Anl Arhgap12, Ermn, Fastkd2, Maoa, Mcm9, Nbeal1, Nf1, Pcgf6, Rffl, Ric3, Sccpdh, Slc18a2, Tm6sf1, Zcchc11
	Npc1$^{-/-}$↓, *Grn*$^{-/-}$↓	47	Rap1a, Them4, Pak3, Idi1, Sc5d, 3632451O06Rik, Brix1, Capza1, Ccny, Fam13a, Glrb, Hspa4, Jam2, Lifr, Lrp1 Luzp2, Lztfl1, Nefh, Opalin, Phip, Plp1, Prepl, Ptpn4, Ralgapa1, Sycp1, Ttc21b, Ube2b, Ublcp1, Usp9x, Ago Cab39, Ccne2, Dnm1l, Erc1, Grb14, Hspa4l, Ipo11, Krit1, Pbrm1, Pkn2, Sass6, Sphkap, Stk17b, Stk39, Strip Suz12, Xpo1
	Npc1$^{-/-}$, *Grn*$^{-/-}$, *Grn*$^{+/-}$	27	Rap1a, Them4, Idi1, 3632451O06Rik, Brix1, Capza1, Ccny, Fam13a, Glrb, Hspa4, Jam2, Lifr, Lrp1b, Luzp2, Lztfl Nefh, Opalin, Phip, Plp1, Prepl, Ptpn4, Ralgapa1, Sycp1, Ttc21b, Ube2b, Ublcp1, Usp9x
**Up-Regulated**	*Grn*$^{-/-}$↑, *Grn*$^{+/-}$↑	48	Tyrobp, Actb, Flnb, Mt2, Polr2f, Mt1, Tecpr1, Ggt7, Plcxd1, Ppt2, 2010300C02Rik, 4930452B06Rik, Cacna1 Cacng8, G530011O06Rik, Gnb5, Greb1l, Hpca, Kcnh3, Atp5k, Celf4, Ckb, Col16a1, Col4a2, Cox6a1, Cox6b Dus3l, Eef1a1, Emc10, Fam107a, Fuz, Gnb2, Hipk4, Mllt6, Ndufa13, Ndufa7, Npy, Pebp1, Plekhb1, Rpl29, Rplp Rps14, Rps19, Rps26, Shisa4, Slc22a17, Slc25a18, Snrpa
	Npc1$^{-/-}$↑, *Grn*$^{+/-}$↑	17	Tyrobp, Elk1, Tuba1a, 2010300C02Rik, 4930452B06Rik, Cacna1h, Cacng8, G530011O06Rik, Gnb5, Greb1l, Hpc Kcnh3, 2410006H16Rik, Camk2n1, Col6a1, Ctxn1, Mast3
	Npc1$^{-/-}$↑, *Grn*$^{-/-}$↑	28	Tyrobp, C1qa, C1qb, C4b, Ctss, H2-D1, Trem2, Cd68, Ctsd, Ftl1, Lyz2, Man2b1, 2010300C02Rik, 4930452B06Ri Cacna1h, Cacng8, G530011O06Rik, Gnb5, Greb1l, Hpca, Kcnh3, Fcrls, Gfap, Gpnmb, Lag3, Mfsd2a, Rasl10b, Xdl
	Npc1$^{-/-}$, *Grn*$^{-/-}$, *Grn*$^{+/-}$	10	Tyrobp, 2010300C02Rik, 4930452B06Rik, Cacna1h, Cacng8, G530011O06Rik, Gnb5, Greb1l, Hpca, Kcnh3

	Genotypes	#	Genes
Opposing Directions	*Grn*$^{-/-}$↑, *Grn*$^{+/-}$↓	1	Gdpd3
	Npc1$^{-/-}$↑, *Grn*$^{+/-}$↓	10	Itgb1, Ap1s2, Grn, Krt12, Scfd1, Serpina3n, Stk38, Tbr1, Vim, Vps50
	Npc1$^{-/-}$↑, *Grn*$^{-/-}$↓	5	Ap1s2, Grn, Erc2, Krt12, Med29
	Npc1$^{-/-}$↑, *Grn*$^{-/-}$ ↓, *Grn*$^{+/-}$↓	3	Grn, Ap1s2, Krt12
	Npc1$^{-/-}$↓, *Grn*$^{+/-}$↑	1	Cycs
	Npc1$^{-/-}$↓, *Grn*$^{-/-}$↑	1	Mid1

Table S5. Related to Figure 5. This table lists the overlapping lysosomal, immune-related, and lipid metabolic genes differentially regulated between *Npc1*- and *Grn*-defici mice. All transcripts with ≥ 1.5-fold change ($p < 0.05$) were considered differentially expressed. Gene associations are color coded: lysosomal function (red), immune respo (green), lipid metabolism (purple), and other (black). Genes similarly changed across genotypes are underlined.

SUPPLEMENTAL EXCEL TABLE LEGENDS

Table S2A-H. Related to Figures 2-4. Mass spectrometry analysis of tissue from the following sources: (S2A-C) human brains with various autopsy-confirmed neurodegenerative diseases (AD, GRN FTLD-TDP, Non-GRN FTLD-TDP, and Control), (S2D-F) wild type and *Grn* mutant mouse brains, (S2G) immortalized wild type and *Grn* mutant mouse embryonic fibroblasts (MEFs), and (S2H) lysosomes enriched from fresh wild type and *Grn* mutant mouse liver. (S2A, D, G, and H) These tables include the precursor m/z ion, the detected m/z fragment, and the corresponding neutral loss (NL) values. Identified lipids are described by name and group: cholesteryl esters (CE); ceramides (Cer); hexosyl ceramides–containing one sugar (HexCer), two sugars (Hex2Cer) or three sugars (Hex3Cer); monoacylglycerides (MAG); diacylglycerides (DAG); triacylglycerides (TAG); sphingomyelins (SM); phosphatidylcholines (PC); phosphatidylethanolamines (PE); phosphatidylglycerols (PG); phosphatidylinositols (PI); phosphatidylglycerols (PG); phosphatidylserines (PS); lysophosphatidylcholine (LPC); and lysophosphatidylethanolamines (LPE). *e* and/or * indicate the presence of an ether bound acyl chain. (S2B and E) These tables list the LDA variables of all the mass features, whether identified or unknown. (S2C and F) This table lists the LDA variables of only the individually identified DAGs, TAGs, PEs, and PSs.

Table S3. Related to Figure 5. This table lists the $\log_2$ fold change ($\log_2$FC) and the corresponding significance (*q* value) of differentially expressed transcripts in 1) $Grn^{+/-}$ and $Grn^{-/-}$ mouse brains as compared to those of $Grn^{+/+}$ mouse brains and 2) $Grn^{-/-}$ mouse brains as compared to those of $Grn^{+/-}$ mouse brains.

Table S4. Related to Figure 5. This table lists the differentially expressed genes between *Npc1*- and *Grn*-deficient mice. All transcripts with ≥ 1.5-fold change ($p < 0.05$) were considered differentially expressed. Gene associations are color coded: lysosomal function (red), immune response (green), lipid metabolism (purple), and other (black). Genes similarly changed across genotypes are bolded and underlined.

SUPPLEMENTAL EXPERIMENTAL PROCEDURES

Generation of *Grn* mutant mice and animal husbandry

The generation of *Grn* mutant ($Grn^{+/-}$ and $Grn^{-/-}$) mice has been previously described (Martens et al., 2012). Animals were maintained on a mixed 129SvEv Bradley;C57BL/6J background by heterozygous intercrossing. Wild type ($Grn^{+/+}$) control mice were obtained from the same crossings. Mice were genotyped by PCR using ear genomic DNA with primers previously described (Martens et al., 2012). Animals were maintained on a 12-hour light/12-hour dark cycle and fed a standard rodent chow diet (Diet 7001; Harlan Teklad, Madison, WI) and water *ad libitum*. No sexual dimorphism of phenotype was observed. All procedures were performed in accordance with the protocols approved by the Institutional Animal Care and Use Committee of the University of Texas Southwestern Medical Center.

Electron Microscopy

Deeply anesthetized 3-month-old *Grn* mutant mice and controls were perfused with PBS via cardiac perfusion. The hippocampus and frontal cortex were dissected from individual mouse brains and placed in 3% glutaraldehyde solution on ice. The fixed tissues were incubated overnight at 4°C. The tissue was then trimmed to small pieces, rinsed in PBS, post-fixed in 1% osmium tetroxide for 1 hour at room temperature, and dehydrated through a graded ethanol series. They were then infiltrated in Eponate 12 resin (Ted Pella, Redding, CA) for 4 hours at room temperature and polymerized overnight at 60°C. Resin blocks were then sectioned at 1.5 μm and stained with toluidine blue for light microscopy. Ultrathin (110 nm) sections were mounted on 200 Iines/inch Hex. Mesh Thin Bar copper grids (Electron Microscopy Sciences, Hatfield, PA) and stained with uranyl acetate and lead citrate. Electron photomicrographs were taken using a Hitachi (Japan) electron microscope at 80kV. Lysosomal size was quantified using the measurement tool software provided by Advanced Microscopy Techniques (Woburn, MA); major and minor axes were defined as the largest and smallest diameters of the lysosome, respectively.

GRN sequencing in human tissue samples

Sequencing of all coding and non-coding exons 1-13 of *GRN* was performed at the University of Texas Southwestern Medical Center (Dallas, TX) and the Mayo Clinic (Jacksonville, FL) for all human subjects included

in this study (Table S1) using DNA extracted from frozen cerebellar tissue as previously described (Baker et al., 2006).

MS sample preparation and lipid extraction

Frozen pieces of fresh, post-mortem human brain were isolated from the cortex of the middle frontal gyrus of autopsy-confirmed cases of AD, FTLD-TDP with and without *GRN* hemizygous loss (GRN FTLD-TDP and Non-GRN FTLD-TDP, respectively), and controls with only age-related neuropathologic changes (Control) (Table S1). Additionally, frozen pieces of fresh hippocampus and frontal cortex from 12-month-old wild type and *Grn* mutant mice were obtained. The frozen brain samples were individually weighed (ranged between 25 and 146 mg), and each was placed in 2 mL pre-filled tubes containing 2.4 mm ceramic beads (Omni^{TM}, Kennesaw, GA) that were previously weighed. Samples were kept frozen in liquid nitrogen to assure integrity during the homogenization process. Samples were then fully homogenized using the Omni ^{TM} Bead-Ruptor 24 (Kennesaw, GA) in 1 mL of 2:1 CH_2Cl_2/MeOH and were then transferred into 16 x 100 mm glass culture tubes where an additional aliquot of 2:1 DCM/MeOH was added to yield a final lipid concentration of 10 mg/mL.

Immortalized pools of *Grn*$^{+/+}$, *Grn*$^{+/–}$, and *Grn*$^{–/–}$ MEFs were used for lipidiomic analysis. Briefly, the harvested cells were pelleted in 16 x 100 mm glass culture tube and were subsequently snap frozen in liquid nitrogen. The cell pellets were lysed in 600 μL of 2:1 DCM/MeOH, vortexed for 5 minutes, and then placed in the ultrasound bath at room temperature for another 10 minutes. Lysosomes from mouse livers were enriched as described below. The lysosome samples were thawed on ice, after which 200 μL of 2:1 DCM/MeOH and 100 μL of PBS were added.

Lipids were extracted using a Bligh-Dyer based liquid-liquid extraction with the help of an automatic Hamilton ^{TM} STARlet Robot (Reno, NV). For human brain samples and MEFs, an aliquot of 500 μL of homogenization solution was transferred to a 2 mL Flexi-Tier ^{TM} glass insert to which was then added an additional 400 μL of 2:1 DCM/MeOH and 300 μL of PBS. For lysosome samples, 300 μL of sample in PBS was transferred to a 2 mL Flexi-Tier ^{TM} glass insert to which was then added 900 μL of 2:1 DCM/MeOH. The samples were then vortexed for 5 seconds and centrifuged at 2500 rpm for 5 minutes. 300 μL of the bottom organic layer was transferred into a new 1 mL Flexi-Tier ^{TM} glass insert and mixed with 300 μL of 2:1 MeOH/Isopropanol (with 14 mM ammonium acetate added). Samples were stored at 4°C in the dark until the MS analysis the same day.

Infusion-based MS/MS^{ALL} analysis, data refining, Linear Discriminant Analysis (LDA) plot generation

70 μL of sample prepared above was directly infused into the electrospray ionizer of a SCIEX 5600+ qTOF mass spectrometer (Redwood City, CA). A Leap Technologies™ PAL-XT (Carrboro, NC) equipped with a 100 μL syringe was used to infuse the sample at 10 μL/min. A non-targeted, infusion-based MS/MS^{ALL} analysis was conducted between 100 to 1200 Da in both positive and negative modes using SCIEX Analyst TF® software. Lipids were named based upon their unique mass combinations and neutral losses (Han and Gross, 2005; Simons et al., 2012). For example, arachidonic acid was identified by its MS2 mass of 303 Da in negative mode or neutral loss of 321 (+NH4) Da in positive mode.

Current SCIEX Analyst® software does not have the capability to refine the complex data set acquired from the MS/MS^{ALL} in a useful way, so a lab self-developed software (using Python programming language) aided in the data processing and cleaning of the MS/MS^{ALL} spectra. Individual peak intensities were divided by the total peak intensity (the sum of all the peaks in a same sample) to normalize by total lipid content. Final values were then reported as relative peak intensity among the total lipid intensity, allowing for a representation of the relative lipid composition within the total lipid pool. These final values do not represent absolute masses or concentrations and are only used to compare different samples with similar characteristics.

To find the optimum group classification and check for similarities between groups, we analyzed the data using LDA on both the human and mouse brain samples. SCIEX MarkerView® software was used to create LDA plots of the raw peak intensities obtained from the mass spectrometer and were normalized to the sum of the total area. These analyses were run with no weighing and using pareto scaling.

Enriched lysosome preparations

Liver harvests were performed in the same manner as described for brain harvests. Lysosome enrichment was performed following a well-established, standard protocol (Graham, 2000). Livers were dissected into two equal amounts, minced into fragments, and added to a homogenization (HM) buffer (0.25 M sucrose, 1mM Na_2EDTA, 10mM HEPES in ddH_2O at pH 7). The tissue was homogenized in 1 mL dounce homogenizers using 15-20 strokes (Figure S2A). Homogenate was transferred to 2 mL Eppendorf tubes and centrifuged in a tabletop centrifuge at 4°C for 10 minutes at 10,000 x g. Supernatant was then removed to a 4 mL high-speed centrifuge tube. Then, 1.5 mL of

HM buffer was added to the Eppendorf and the pellet was re-suspended with a 16-gauge needle. Centrifugation and supernatant removal was repeated. The combined supernatants were then centrifuged at 20,000 x g in a Sorvall RC centrifuge with fixed angle rotor S100AT6 with acceleration and deceleration at top speed. Supernatant was discarded, and the pellet was re-suspended in 3 mL of HM buffer. Centrifugation and supernatant discard was repeated. The remaining pellet was then re-suspended in 1 mL of HM buffer and 550 μL of the mixture was added to a 1 mL ultracentrifuge tube. Percoll was added to Percoll diluent (2.5 M sucrose, 10mM Na_2EDTA, 100mM HEPES, at pH 7) to make a stock Percoll solution of 90% (v/v) Percoll. 450 μL of stock solution added to each 1 mL tube of liver preparation. Tubes were placed in S120AT2 fixed-angle rotor and centrifuged at 35,000 x g for 1.5 hours at quickest acceleration setting and slowest deceleration setting. The resultant layers were then transferred into separate 1 mL ultracentrifuge tubes and centrifuged at 100,000 x g for one hour at quickest acceleration and deceleration setting. This spin pelleted the remaining Percoll, and supernatant was removed for further testing.

Overall, these enrichment procedures, which primarily use differential density centrifugation, generally cannot produce homogeneously pure organellar fractions. However, they greatly concentrate subpopulations of organelles into defined fractions, resulting in this case in fractions that not only contained the majority of the cells' lysosomes, but also peroxisomes and some portions of plasma membrane (Figure S2D). For the purpose of the experiment, which primarily aimed at removing the primary source of irrelevant lipid contamination, *i.e.* hepatocytic lipid droplets, the minor contribution of other cellular organelles is inconsequential.

PGRN antibody generation

Rabbit polyclonal antibodies were generated against Linker-1 mouse progranulin peptide containing an amino-terminal additional cysteine for maleimide coupling (CTLLKKFPAQKTNRAVSL, amino acid residues 185 through 201) and an amidated carboxyl terminus conjugated to keyhole limpet hemocyanin (KLH). Briefly, 2 mg progranulin peptide was dissolved in 1 mL of MilliQ water, added to 2 mg lyophilized maleimide-activated KLH, and incubated at room temperature for 2 hours. New Zealand White rabbits were immunized with this conjugated peptide solution.

Immunoblotting

Lysosome-enriched subcellular organellar preparations were diluted 1:2 in MillQ water, and 6x sample buffer was added. All samples were then boiled at 85ºC for 10 minutes and cooled in ice. Samples were loaded at 12.5 μL per well on a 4-12% Tris glycine gel and separated using SDS-PAGE. Proteins were then transferred to nitrocellulose membranes, which were subsequently blocked using 5% BSA in PBS with 0.1% Tween20 (PBST) for one hour. Membranes were placed in primary antibody solution and incubated on a rocking shaker overnight at 4ºC. All primary antibodies were diluted in 5% BSA in PBST. Antibodies were used at the following dilutions: affinity purified linker-1 anti-progranulin antibody at 1:5000, anti-NCP1 at 1:1000, anti-LAMP1 (BD Biosciences, San Jose, CA) at 1:2000, anti-cathepsin D (Abcam, Cambridge, MA) at 1:4000, anti-EEA1 (Cell Signaling, Danvers, MA) at 1:1000, anti-Na^+/K^+ ATPase (Cell Signaling, Danvers, MA) at 1:1000, anti-ACAT at 1:2000, anti-Gm130 at 1:1000, and PMP70 at 1:1000. IRDye infrared fluorescent dye-labeled anti-rabbit, anti-mouse, and anti-goat secondary antibodies (LI-COR Biosciences, Lincoln, NE) were used at a dilution of 1:20,000 to visualize bands. Membranes were scanned and imaged with Odyssey infrared imager (LI-COR Biosciences). All band signals were quantified using Image Studio (LI-COR Biosciences) and normalized to protein levels.

mRNA sequencing from *Grn* mutant mouse brains

Whole brains were dissected from $Grn^{+/+}$, $Grn^{+/–}$, and $Grn^{–/–}$ female mice (n = 2 per genotype; average age ± SEM = 200 ± 23-days-old) and stored in RNAlater (Ambion™, Thermo Fisher Scientific, Waltham, MA) at 4°C until extraction. Brains were homogenized in Trizol (Ambion™), and total RNA was extracted according to the manufacturer's protocol and stored in Rnase-free water at 80°C. RNA was quality checked with the Agilent 2100 Bioanalyzer (average RNA-integrity, Rin, 8.77 ± 0.03). Two mRNA libraries were prepared from each genotype with the TruSeq Stranded mRNA Sample Preparation Kit by the UT Southwestern McDermott sequencing core. All six mRNA libraries (single-end, 50-base pair reads) were run on one flow-cell lane and sequenced with the Illumina Hiseq 2500 v3 sequencing system. Data were analyzed by the UT Southwestern McDermott bioinformatics core.

Illumina CASAVA 1.8.1 was used for base-calling and de-multiplexing with default parameters, and reads were compiled into Fastq files and filtered for quality (average number of mapped reads across samples were 23.42 ± 1.17 million mapped reads). The reads were then mapped to the mouse genome (NCBI m37/mm9) with Bowtie and Tophat. Expression was then normalized with Cufflinks and featureCounts, and statistical comparisons between

the genotypes were performed with Cuffdiff (Trapnell et al., 2012). Transcripts with > 1.5-fold change ($q < 0.05$) were considered differentially expressed.

Venn diagrams and comparison of differentially expressed gene lists were created with the web-based Area-Proportional Venn Diagram Plotter by BxToolBox (http://apps.bioinforx.com/bxaf7c/app/venn/app_overlap.php). A network diagram was created with web-based STRING 10.0 database (Szklarczyk et al., 2015). Lysosomal genes were obtained from The Mouse Lysosome Gene Database (http://lysosome.unipg.it/mouse.php). Immune system genes were compiled from the Reactome Immune System pathway (R-MMU-168256). Lipid metabolic genes used for the comparisons are detailed as previously described (Zhang et al., 2013). Heat maps were created with the web-based GENE-E module (http://www.broadinstitute.org/cancer/software/GENE-E/index.html) using GenePattern.

The NPC1 microarray dataset (GSE39621) was analyzed with GEO2R (http://www.ncbi.nlm.nih.gov/geo/geo2r/?acc=GSE39621) (Alam et al., 2012) (samples analyzed with GEO2R: *Npc1*$^{+/+}$, 60-67 days old, GSM973252, GSM973253, GSM973258; *Npc1*$^{-/-}$, 59-84 days old, GSM973256, GSM973257, GSM973260, GSM973263, GSM973264) and the transcripts with > 1.5-fold change ($p < 0.05$) were considered differentially expressed. The up- and down-regulated transcripts were compared to the genes regulated in the *Grn* mutant mice.

SUPPLEMENTAL REFERENCES

Chung J-K., Kang, H-S., Lee, C., et al. (1997). Synaptojanin inhibition of phospholipase D activity by hydrolysis of phosphatidylinositol 4,5-bisphosphate. Journal of Biological Chemistry *272*(25), 15980-15985.

Gellatly, S. A., Kalujnaia, S., & Cramb, G. (2012). Cloning, tissue distribution and subcellular localization of phospholipase C X-domain containing protein (PLCXD) isoforms. Biochemical and Biophysical Research Communications *424*(4), 651-656.

Han, X., and Gross, R.W. (2005). Shotgun lipidomics: Electrospray ionization mass spectrometric analysis and quantitation of cellular lipidomes directly from crude extracts of biological samples. Mass Spectrom. Rev. *24*, 367–412.

Irvine, R. F. (2016). A short history of inositol lipids. Journal of Lipid Research *57*, 1987-1994.

Nelson, R. K., & Frohman, M. A. (2015). Physiological and pathophysiological roles for phospholipase D. Journal of Lipid Research *56*(12), 2229-2237.

Ohshima, N., Kudo, T., Yamashita, Y., Mariggiò, S., Araki, M., Honda, A., et al. (2015). New Members of the Mammalian Glycerophosphodiester Phosphodiesterase Family. Journal of Biological Chemistry *290*(7), 4260-4271.

Sato, S.-I., Inoue, H., Kogure, T., Tagaya, M., & Tani, K. (2010). Golgi-localized KIAA0725p regulates membrane trafficking from the Golgi apparatus to the plasma membrane in mammalian cells. FEBS Letters *584*(21), 4389-4395.

Simons, B., Kauhanen, D., Sylvänne, T., Tarasov, K., Duchoslav, E., and Ekroos, K. (2012). Shotgun Lipidomics by Sequential Precursor Ion Fragmentation on a Hybrid Quadrupole Time-of-Flight Mass Spectrometer. Metabolites *2*, 195–213.

Szklarczyk, D., Franceschini, A., Wyder, S., Forslund, K., Heller, D., Huerta-Cepas, J., Simonovic, M., Roth, A., Santos, A., Tsafou, K.P., et al. (2015). STRING v10: protein-protein interaction networks, integrated over the tree of life. Nucleic Acids Research *43*, D447–D452.

Tokumura, A., Majima, E., Kariya, Y., Tominaga, K., Kogure, K., Yasuda, K., & Fukuzawa, K. (2002). Identification of Human Plasma Lysophospholipase D, a Lysophosphatidic Acid-producing Enzyme, as Autotaxin, a Multifunctional Phosphodiesterase. Journal of Biological Chemistry *227*(42), 39436-39442.

Trapnell, C., Roberts, A., Goff, L., Pertea, G., Kim, D., Kelley, D.R., Pimentel, H., Salzberg, S.L., Rinn, J.L., and Pachter, L. (2012). Differential gene and transcript expression analysis of RNA-seq experiments with TopHat and Cufflinks. Nat Protoc *7*, 562–578.

Zhang, Y., Zou, X., Ding, Y., Wang, H., Wu, X., and Bin Liang (2013). Comparative genomics and functional study of lipid metabolic genes in Caenorhabditis elegans. BMC Genomics *14*, 164-177.
